# Supplementary material for: Primitive noble gases sampled from ocean island basalts cannot be from the Earth’s core
Source: Nat Commun. 2022 Jun 30;13:3770. doi: 10.1038/s41467-022-31588-7 (PMC9247082; doi:10.1038/s41467-022-31588-7)
Supplement: Supplementary file 1 — Supplementary Information [file 41467_2022_31588_MOESM1_ESM.pdf]

Supplementary Information for

Primitive noble gases sampled from ocean island basalts cannot be from the Earth's core

Yunguo Li[1,2,3*], Lidunka Vočadlo[1], Chris Ballentine[4], John P. Brodholt[1,5]

[1]*Department of Earth Sciences, University College London, Gower Street, London WC1E 6BT, United Kingdom*

[2]*CAS Key Laboratory of Crust-Mantle Materials and Environments, School of Earth and Space Sciences, University of Science and Technology of China, Hefei 230026, China*

[3]*CAS Center for Excellence in Comparative Planetology, School of Earth and Space Sciences, University of Science and Technology of China, Hefei 230026, China*

[4]*Department of Earth Sciences, University of Oxford, South Parks Road, Oxford OX1 3AN, UK*

[5]*Centre for Earth Evolution and Dynamics, University of Oslo, Oslo, Norway*

This PDF file includes:

Supplementary Methods

Supplementary Discussion

Supplementary Figure 1: Free energies and chemical potentials. Gibbs free energy $\bar{G}$ of **a**) $Fe_{1-x}He_x$ and **b**) $(MgSiO_3)_{1-x}He_x$ melts, and derived $\bar{\mu}_{\mathrm{He}}$ quantities in **c**) iron and **d**) silicate melts.

Supplementary Figure 2: Alchemical thermodynamic integration. Calculated integrands for alchemical thermodynamic integration free energy calculations.

Supplementary Figure 3: Concentration dependence of partition coefficient. Noble gas partition coefficients between liquid iron and silicate melt as a function of noble gas molar fraction in $(MgSiO_3)_{1-x}X_x$.

Supplementary Figure 4: He partition coefficient. Comparison between our He partition coefficients with literature partition coefficients (in weight) between liquid Fe and silicate melt. Note that the results of Xiong *et al*. and Wang *et al*. have been obtained using their chemical potentials but corrected as discussed in S2.

Supplementary Figure 5: Ar partition coefficient. Comparison between our Ar partition coefficients with literature partition coefficients (in weight) between liquid Fe and silicate melt. As with Fig. S4, the *D*s of Xiong *et al*. and Wang *et al*. have been corrected using the methods described in S2.

Supplementary Figure 6: Comparison of chemical potentials. The chemical potential difference between in liquid Fe and in silicate melt from Wang *et al*. and this study.

Supplementary Figure 7: Comparison of partition coefficients. Comparison between our partition coefficients and those of Wang *et al*. 2022.

Supplementary Table 1: Chemical potential ($\bar{\mu}$) difference between in liquid iron and in silicate melt (in eV/atom).

Supplementary Table 2: Fitted parameters for Lennard-Jones model $\bar{\mu}_{\mathrm{X}}^{\mathrm{Fe}} - \varepsilon \cdot \left[\left(\frac{r_0}{r}\right)^{12} - 2\left(\frac{r_0}{r}\right)^{6}\right]$.

Supplementary Table 3: Abundances of primordial noble gas nuclides in the core. ^{3}He budget in the bulk silicate Earth (BSE) is from nebular ingassing model that assumes a 10 Myr accretion time and 1 yr mean surface age[13]. Abundances of ^{22}Ne, ^{36}Ar and ^{130}Xe in BSE were calculated based their relative concentration to ^{3}He in ocean island basalts[14]. The ^{78}Kr abundance in BSE was calculated according to its relative abundance to ^{130}Xe in the whole Earth[15]. Abundances of the other nuclides were obtained by calibrating to their isotope solar ratios[16].

Supplementary Methods

Noble gas has a negligible solubility in liquid iron at room pressure[1]. It is not clear how this will change with pressure, but it is unlikely to have an appreciable solubility up to the core-mantle differentiation pressure. The pure component of helium chemical potential $\bar{\mu}(p, T, x)$ is the same for both an infinitely diluted solution and a much denser solution, only if the local coordination environment of helium is the same. This allows us to simulate the solution in a supercell of limited size. We put one helium atom in supercells with 48, 64, 72, 96 and 108 Fe atoms, respectively, and calculated the helium chemical potential. As we go to the thermodynamic limit in free energy calculations, we find that there is no measurable size effect on the chemical potential. We estimated the uncertainty of helium chemical potential to be less 0.1 eV from the size effect. We also calculated the He chemical potential for more concentrated solutions by adding two and three He atoms to a supercell of 64 Fe atoms and two/three He atoms. From analysis of the RDF and coordination number, there is a slight pairing of He atoms in these more concentrated solutions, and in the extreme case He is partially coordinated to He in $Fe_{64}He_3$. The He cluster in solid Fe has a binding energy of ~0.2 eV/He atom[2], and this will be less in liquid due to the reduced strain effect[3]. So, we may have underestimated the He chemical potential by ~0.2 eV in these highly concentrated $Fe_{64}He_2$ and $Fe_{64}He_3$ systems, which will become smaller when averaged with other compositions. We also performed alchemical free energy calculations at 20 GPa and 50 GPa by changing H to He in $Fe_{64}H_2$, and obtained the chemical potential differences between H and He, and by using the reported H chemical potentials of H[4] we obtained another estimate for the He

chemical potentials. The new chemical potentials agree well with the ones above, indicating that they are robust.

Noble gas solubility is relatively much higher in silicate melts, and for helium it can reach up to 10% in molar fraction from 10 to 100 GPa[5,6]. We calculated the helium chemical potential in supercells with 24, 32, 40, and 48 units of $MgSiO_3$, respectively. With four He atom in the supercell of $(MgSiO_3)_{32}He_4$, there is little sign of clustering of He atoms. This agrees with the He solubility in silicate melts from previous studies[5,6]. The calculated Gibbs free energies and chemical potentials were plotted in Fig. S1.

For the alchemical free energy calculation, the chemical potential difference between noble gas X and He can be rewritten as

$$\mu_{\mathrm{XHe}} = -k_B T \ln \frac{N_{\mathrm{He}}}{N_{\mathrm{X}}+1} - k_B T \ln \frac{\Lambda_{\mathrm{He}}^3}{\Lambda_{\mathrm{X}}^3} - k_B T \ln \left\{ \frac{\int_V d\boldsymbol{R} \exp\left[-\frac{U(N_{\mathrm{He}}-1, N_{\mathrm{X}}+1)}{k_B T}\right]}{\int_V d\boldsymbol{R} \exp\left[-\frac{U(N_{\mathrm{He}}, N_{\mathrm{X}})}{k_B T}\right]} \right\} =$$

$$-k_B T \ln \frac{N_{\mathrm{He}}}{N_{\mathrm{X}}+1} - k_B T \ln \frac{\Lambda_{\mathrm{He}}^3}{\Lambda_{\mathrm{X}}^3} + \int_0^1 d\lambda \langle U_1 - U_0 \rangle_\lambda \qquad (1)$$

Where $U_1(\boldsymbol{R}) = U(N_{\mathrm{He}} - 1, N_{\mathrm{X}} + 1)$ and $U_0(\boldsymbol{R}) = U(N_{\mathrm{He}}, N_{\mathrm{X}})$, and the thermal average $\langle \cdot \rangle$ can be calculated in the system under thermal equilibrium governed by the energy function $U_\lambda = (1 - \lambda) U_0 + \lambda U_1$. The integration takes five to seven points to reach convergence in this study, depending on the difference of atom size between He and X. We used supercells of $Fe_{108}He_2$ and $(MgSiO_3)_{32}He_2$ for liquid iron and silicate melt, respectively. One He atom was gradually converted to other noble gas element. The calculated integrands U_λ are plotted in Fig. S2.

AIMD calculations were run at the density functional theory (DFT) level with the projector augmented wave (PAW) method[7,8] using the VASP code[9,10]. Exchange-correlation effects were treated with the generalized gradient approximation (GGA)

parameterized of Perdew, Burke and Ernzerhof[11]. The Fermi–Dirac statistics was used to populate single particle orbitals. The plane-wave basis was generated with valence configurations of Fe-$3p^6 3d^7 4s^1$, Mg-$2p^6 3s^2$, Si-$3s^2 3p^2$, O-$2s^2 2p^4$, He-$1s^2$, Ne-$2s^2 2p^6$, Ar-$3s^2 3p^6$, Kr-$4s^2 4p^6$, Xe-$5s^2 5p^6$. The energy is converged to within 1 meV/atom and pressure within 0.15 GPa together with a 2 × 2 × 2 Monkhorst-Pack k-mesh for a plane-wave energy cutoff of 600 eV and 800 eV for the iron and silicate melts, respectively. We first ran the calculations using a single Gamma point and an energy cutoff of 400 eV and 600 eV for the iron and silicate melts, respectively, and then corrected the energies by doing thermodynamic integration to 600 eV and 800 eV, respectively[12,13], to save on computer time. We relaxed the iron and silicate melts at 50 GPa (3500 K) and 135 GPa (4200 K) with constrained NPT calculations that maintained the cubic supercell. NPT calculations were run for over 10 *ps* and the lattice parameters were averaged from the part excluding at least the first 2 *ps*. These derived lattice parameters were then used for NVT calculations, and sufficient long time was used to obtain satisfying statistics and the first picoseconds were discarded.

The uncertainties in MD energies were obtained using the standard blocking method and integrated to obtain the uncertainties of free energies and chemical potentials following the error propagation rule. The uncertainty in free energy from our AIMD simulations comes from two parts: one is that of volume under target pressure and the other is from fluctuation of potential energy. The later term is typically 5 meV/atom[12,14], and the first term is normally no less than the last term[14]. Our uncertainties in free energy are typically ~5-10 meV/atom.

Supplementary Discussion

Two similar studies using density functional calculations on noble gas partitioning have been published recently, which show some inconsistency with our results. Xiong *et al*. calculated the partition coefficients of He and Ar between liquid iron and silicate melt at various pressures[15], while Wang *et al*. obtained partition coefficients for He, Ne, Ar, Kr and Xe under pressures from 10 to 135 GPa[16]. After careful examination, we find the inconsistency originates from both the uncertainty of free energy calculation and also the thermodynamic treatment of partition coefficient.

As we have explained above, the chemical potential is a derivative of the system Gibbs free energy. Therefore, the uncertainty of chemical potential reduces with the increasing number of concentrations considered. We considered four to five compositions in the free energy calculations to improve the statistics. Xiong *et al*. on the other hand, calculated the Gibbs free energy change for this reaction only

$$((MgSiO_3)_{16}+X_2) + Fe_{50} = (MgSiO_3)_{16} + (Fe_{50}+X_2) \quad (2)$$

This is equivalent to

$$((MgSiO_3)_{16}+X_2) - (MgSiO_3)_{16} = (Fe_{50}+X_2) - Fe_{50} \quad (3)$$

In this case the free energy change in the left-hand side is equal to twice of the chemical potential of X in silicate melt, and the free energy change in the right-hand side is equal to twice of the chemical potential of X in liquid Fe. Thus, the chemical potential is actually obtained from one single composition. Similarly, Wang *et al*. calculated the Gibbs free energy change for this reaction

$$((MgSiO_3)_{16}+X) + Fe_{64} = (MgSiO_3)_{16} + (Fe_{64}+X) \quad (4)$$

Their chemical potential is also obtained from a single composition free energy calculation. Since the uncertainty in the energy in any calculated free energy is about 0.01 eV/atom, then, the uncertainty in the noble gas chemical potential in iron would be approximately $64 \times 0.01 \times \sqrt{2}$ eV, calculated in a supercell of 64 atoms. Similarly, the uncertainty in the noble gas chemical potential in silicate is ~$80 \times 0.01 \times \sqrt{2}$ eV, calculated in a supercell of 80 atoms. This leads to an uncertainty of three orders of magnitude in *D*. The uncertainties of the chemical potentials can be significantly reduced by considering more concentrations, as we have done. For some reason both studies report very low chemical potential/free energy uncertainties which we note are significantly less than in other comparable studies[12,14].

We also note that the chemical potential difference of He between in liquid iron and in silicate melt at 135 GPa and 5000 K is reported to be 3.13 eV by Xiong *et al*. and 1.67 eV by Wang *et al*. This very large difference of 1.46 eV between the two studies for the same system using the same method is much larger than their estimates of uncertainty, which are 0.07 eV and 0.32 eV, respectively, but much more understandably if the uncertainties are similar to those we estimate in the previous paragraph.

The other aspect that distinguishes our method with theirs is the thermodynamic treatment of the partition coefficient. We have detailed our procedure in a previous publication[4], and now we review it in more detail here. Starting with the Gibbs free energy at any P, T and x,

$$G(x) = \bar{G}(x) - k_B T \cdot S_{mix}(x) \qquad (5)$$

where $\bar{G}(x)$, the ***excess*** free energy, is the difference between the total Gibbs free energy and the ***ideal*** entropy of mixing $S_{mix}(x)$. This $\bar{G}(x)$ is exactly what we obtain from

thermodynamically integrating from an ideal gas. From Gibbs-Duhem equation we can then derive the chemical potential in the normal way as

$$\mu_X(x) = G(x) + (1-x)\frac{\partial G(x)}{\partial x} \tag{6}$$

Substituting $G(x)$ from Eq. (5) into the above equation we get

$$\mu_X(x) = \bar{G}(x) + (1-x)\frac{\partial \bar{G}(x)}{\partial x} - k_B T(S_{mix}(x) + (1-x)\frac{\partial S_{mix}(x)}{\partial x}) \tag{7}$$

or

$$\mu_X(x) = \bar{\mu}_X(x) - k_B T(S_{mix}(x) + (1-x)\frac{\partial S_{mix}(x)}{\partial x}) \tag{8}$$

Leaving aside the $\bar{\mu}_X(x)$ terms for the moment, and focusing first on the Fe system,

$$S_{mix}(x) = x\ln(x) + (1-x)\ln(1-x) \tag{9}$$

and so

$$S_{mix}(x) + (1-x)\frac{\partial S_{mix}(x)}{\partial x} = \ln(x) \tag{10}$$

and by substituting Eqs. (9) and (10) into (8) the chemical potential of noble gas in Fe becomes just

$$\mu_X(x) = \bar{\mu}_X(x) + k_B T\ln(x) \tag{11}$$

Note that the non-ideal mixing entropy is subsumed into the first term, $\bar{\mu}_X(x)$.

We can do the same for the silicate melt, however, the entropy of mixing is somewhat more complicated since it is mixing of three components in the $MgSiO_3$ silicate and one component of noble gas. In this case the ideal entropy of melting is

$$S_{mix}(y) = y\ln\left(\frac{y}{5-4y}\right) + 2(1-y)\ln\left(\frac{1-y}{5-4y}\right) + (3-3y)\ln\left(\frac{3-3y}{5-4y}\right) \tag{12}$$

Nevertheless, taking the derivative again and with some manipulation, the chemical potential of a noble gas in the silicate simplifies to

$$\mu_X(y) = \bar{\mu}_X(y) + k_B T \ln\left(\frac{y}{5-4y}\right) \tag{13}$$

where again the non-ideality of mixing is wrapped into the first two terms.

Finally, equating the chemical potential of a noble gas, X, in Fe with the chemical potential in the silicate we get

$$\bar{\mu}_X(x) + k_B T \ln(x) = \bar{\mu}_X(y) + k_B T \ln\left(\frac{y}{5-4y}\right) \tag{14}$$

and

$$e^{-\frac{\bar{\mu}_X(x) - \bar{\mu}_X(y)}{k_B T}} = \frac{x(5-4y)}{y} \tag{15}$$

Since y is vey small (i.e. order 10^{-10}), then $(5 - 4y) \cong 5$ and we obtain D in weight fraction

$$D_{wt.} = 1.78D = 1.78\,\frac{x}{y} = \frac{1.78}{5} e^{-\frac{\bar{\mu}_X(x) - \bar{\mu}_X(y)}{kT}} \tag{16}$$

where the factor of 1.78 is 100/56, the ratio of molar weights of $MgSiO_3$ to Fe.

So far there is no loss in generality, nor any assumption of ideal-mixing since the $\bar{\mu}_X$terms in Eq. (16) contain any non-ideality. This is equivalent to definition of D given in Bennett (2021)[17], which would give

$$-\Delta G^{\circ} = k_B T \cdot \ln\left(\frac{a^{Fe}}{a^{MgSiO_3}}\right) = k_B T \cdot \ln\left(\frac{\gamma_X^{Fe} \cdot x}{\gamma_X^{MgSiO_3} \cdot \frac{y}{5-4y}}\right) \tag{17}$$

and,

$$-\Delta G^{\circ} - k_B T \cdot \ln\left(\frac{\gamma_X^{Fe}}{\gamma_X^{MgSiO_3}}\right) = k_B T \cdot \ln\left(\frac{x(5-4y)}{y}\right) \quad (18)$$

Finally, *D* is given as

$$D = \frac{x}{y} = \frac{1}{5}\frac{\gamma_X^{Fe}}{\gamma_X^{MgSiO_3}} \cdot e^{\frac{-\Delta G^{\circ}}{k_B T}} \quad (19)$$

where ΔG° is the standard Gibbs free energy of reaction for the transfer of solute between the pure phases of liquid Fe and silicate melt. and has no mixing entropy contribution. Non-ideality is taken care by the activities a^{Fe} and a^{MgSiO_3}. γ_X^{Fe} and $\gamma_X^{MgSiO_3}$ are the activity coefficients.

Eqs. (16) and (19) are thermodynamically equivalent under either ideal or non-ideal circumstances. They only differ in the treatment of non-ideality. We find the calculated Gibbs free energies are justified with an ideal gas mixing model (Fig. S2). Then the activity coefficients are equal to one, ΔG° in Eq. (19) is equivalent to $\bar{\mu}_X(x) - \bar{\mu}_X(y)$, and Eqs. (16) and (19) become identical.

On the other hand, both Xiong *et al*. and Wang *et al*. obtained the partition coefficient from the equilibrium constant (*K*) for the reactions (2) and (4). Xiong *et al*. expressed

$$K = exp\left(\frac{-\Delta G}{k_B T}\right) = \frac{x(1-y)}{y(1-x)} \quad (20)$$

That is

$$\Delta G = G(Fe_{50}X_2) - G(Fe_{50}) - (G((MgSiO_3)_{16}X_2) - G((MgSiO_3)_{16})) = -k_B T \cdot \ln\left(\frac{x(1-y)}{y(1-x)}\right) \quad (21)$$

and equivalently

$$[G(Fe_{50}X_2) + k_BT \cdot \ln(x)] - [G(Fe_{50}) + k_BT \cdot \ln(1-x)]$$

$$=$$

$$[G((MgSiO_3)_{16}X_2) + k_BT \cdot \ln(y)] - [G((MgSiO_3)_{16}) + k_BT \cdot \ln(1-y)] \tag{22}$$

It appears that they used full Gibbs free energies Gs already containing the mixing entropy that should have been removed. In addition, here x and y would correspond to the concentration of $Fe_{50}X_2$ in the Fe-$Fe_{50}X_2$ system the concentration of $(MgSiO_3)_{16}X_2$ in the $MgSiO_3$-$(MgSiO_3)_{16}X_2$ system, which would be a crude approximation to the mixing entropy. Wang *et al*. used a further unusual expression of mixing entropy term.

Nonetheless, we used the calculated free energies from Xiong *et al*. and Wang *et al*. to derive the partition coefficients they should have obtained using our procedure. The partition coefficients for He and Ar are plotted in Figs. S4 and S5. For He, we find the corrected D_{He} of Xiong *et al*. match ours at 50 GPa and 135 GPa, but differ with an order of magnitude at 20 GPa. The corrected D_{He} of Wang *et al*. match ours only at 50 GPa. For Ar, D_{Ar} of Xiong *et al*. match ours at 50 GPa and 135 GPa, but D_{Ar} of Wang *et al*. match ours only at 50 GPa. This mismatch is consistent with the large uncertainties in their chemical potentials, as explained above.

Nevertheless, since Wang *et al*. have performed the calculations for all the five noble gases, it is useful to compare the trend in D for the different noble gases. We plotted chemical potential difference and partition coefficients in Figs. S6 and S7. We observe that their trending is similar to ours. Importantly, the chemical potential

difference is the largest for Ne, and reduces on both sides. This results in a partition coefficient that is the smallest for Ne and increases on both sides. As well, we note that the partition coefficient of Ne is at least two orders of magnitude lower than He, again consistent with our results.

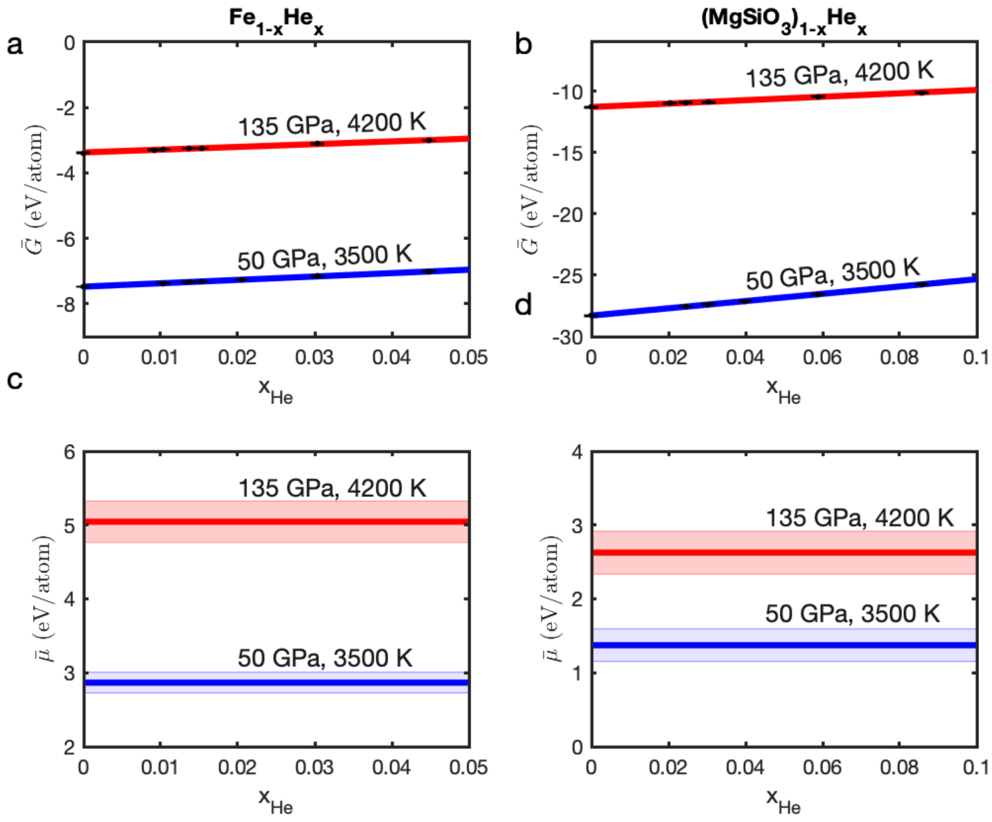

Supplementary Figure 1: Free energies and chemical potentials. Gibbs free energy $\bar{G}$ of **a**) $Fe_{1-x}He_x$ and **b**) $(MgSiO_3)_{1-x}He_x$ melts, and derived $\bar{\mu}_{He}$ quantities in **c**) iron and **d**) silicate melts.

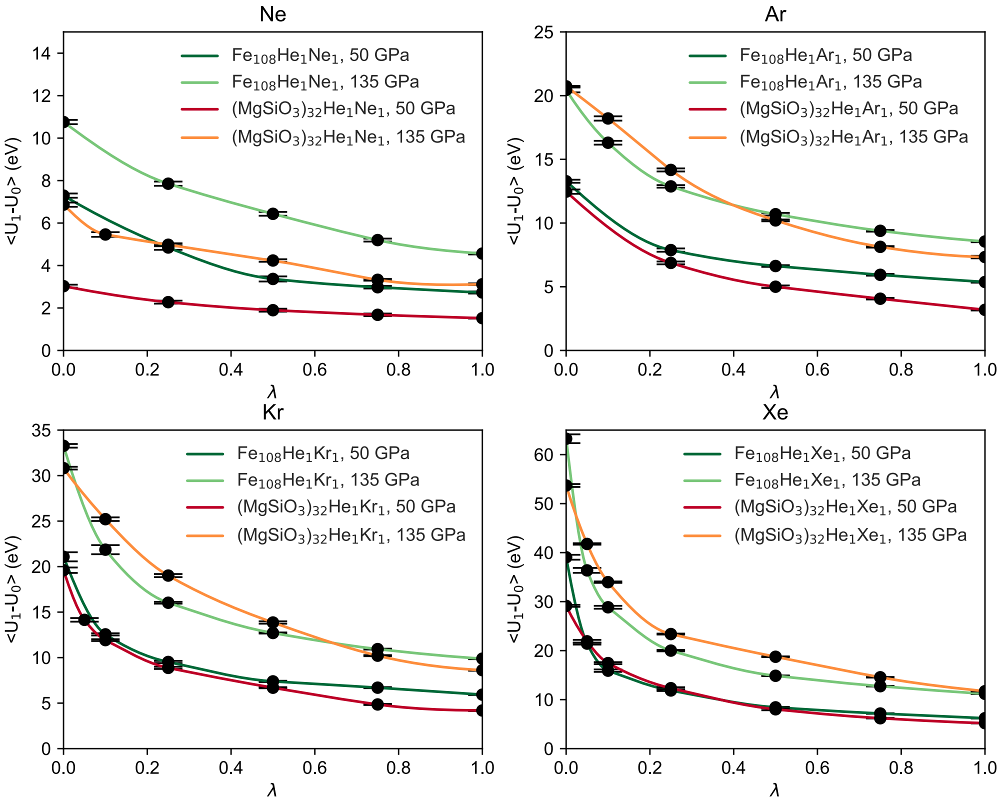

Supplementary Figure 2: Alchemical thermodynamic integration. Calculated integrands for alchemical thermodynamic integration free energy calculations.

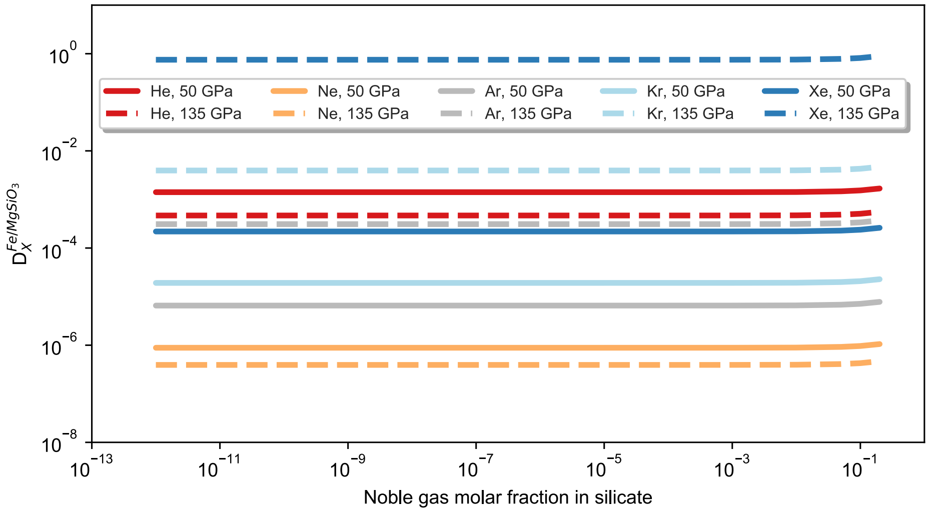

Supplementary Figure 3: Concentration dependence of partition coefficient. Noble gas partition coefficients between liquid iron and silicate melt as a function of noble gas molar fraction in $(MgSiO_3)_{1-x}X_x$.

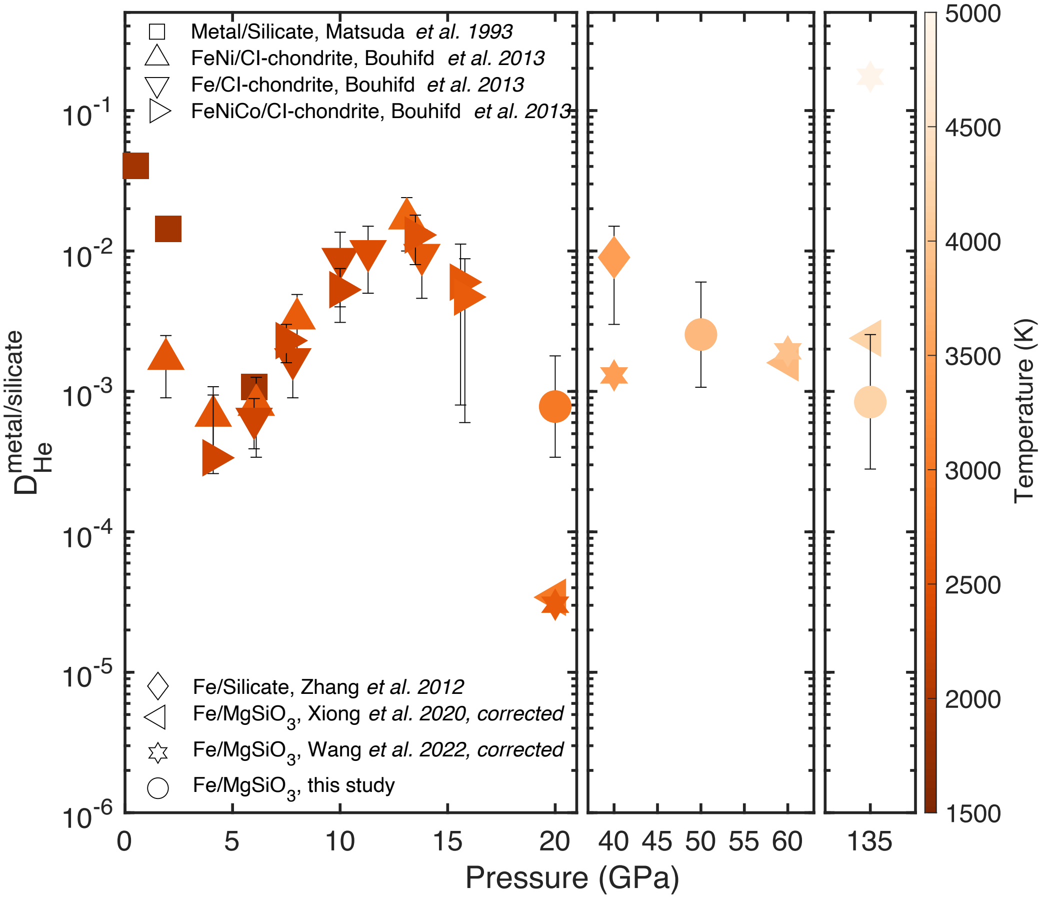

Supplementary Figure 4: He partition coefficient. Comparison between our He partition coefficients with literature partition coefficients (in weight) between liquid Fe and silicate melt. Note that the results of Xiong *et al*. and Wang *et al*. have been obtained using their chemical potentials but corrected as discussed in S2.

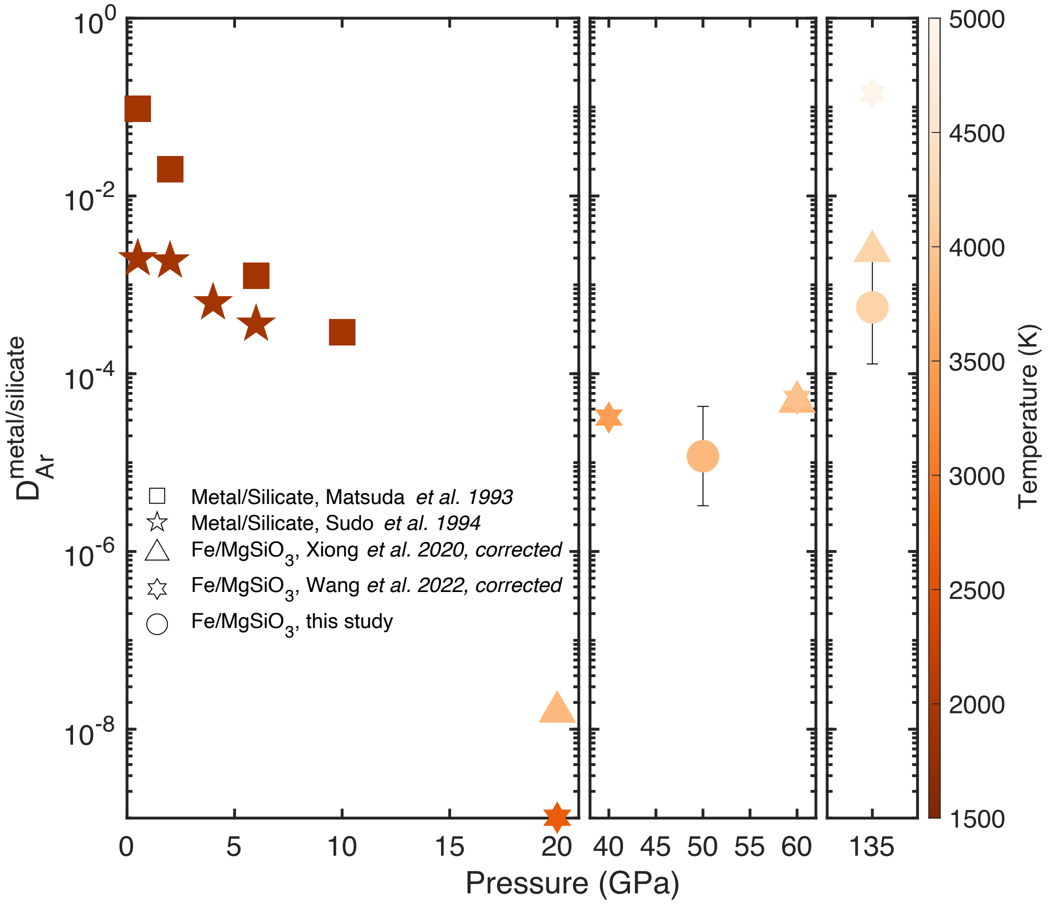

Supplementary Figure 5: Ar partition coefficient. Comparison between our Ar partition coefficients with literature partition coefficients (in weight) between liquid Fe and silicate melt. As with Fig. S4, the *D*s of Xiong *et al*. and Wang *et al*. have been corrected using the methods described in S2.

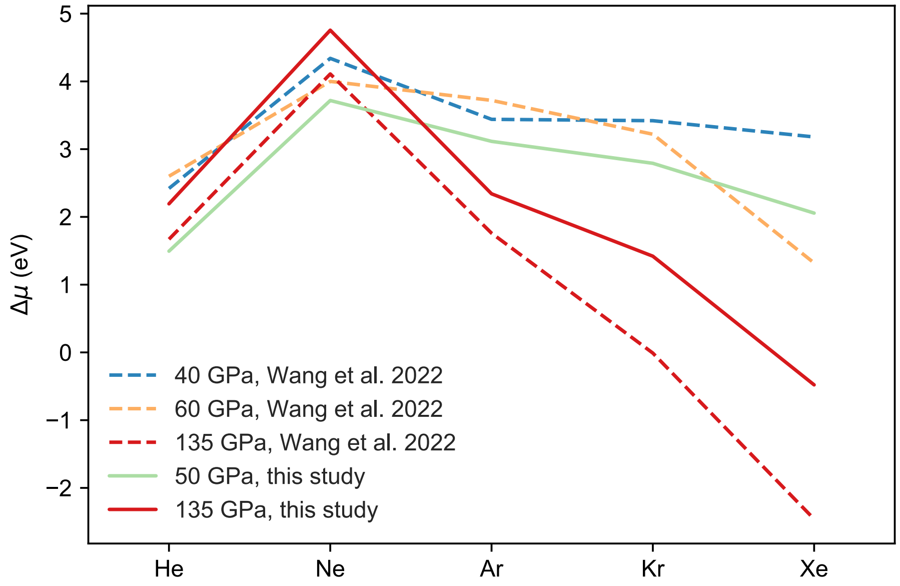

Supplementary Figure 6: Comparison of chemical potentials. The chemical potential difference between in liquid Fe and in silicate melt from Wang *et al*. and this study.

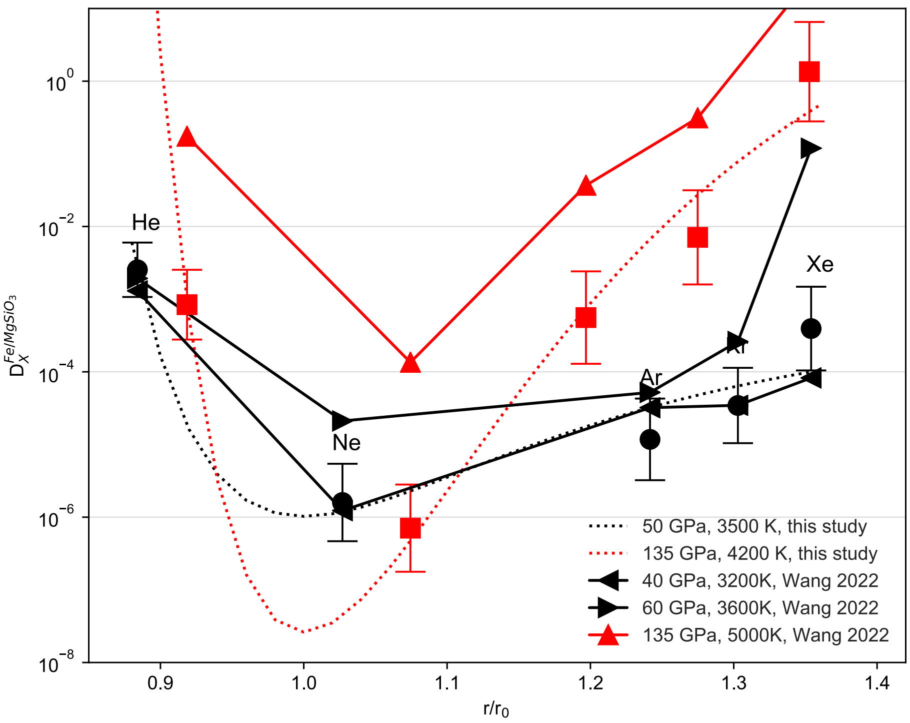

Supplementary Figure 7: Comparison of partition coefficients. Comparison between our partition coefficients and those of Wang *et al*. 2022.

Supplementary Table 1: Chemical potential ($\bar{\mu}$) difference between in liquid iron and in silicate melt (in eV/atom).

P (GPa)	He	Ne	Ar	Kr	Xe
50	1.494	3.718	3.115	2.791	2.056
135	2.193	4.756	2.341	1.421	-0.479

Supplementary Table 2: Fitted parameters for Lennard-Jones model $\bar{\mu}_X^{Fe} - \varepsilon \cdot \left[\left(\frac{r_0}{r}\right)^{12} - 2\left(\frac{r_0}{r}\right)^{6}\right]$.

P (GPa)	T (K)	$\bar{\mu}_X^{Fe}$ (eV)	ε (eV)	r_0 (Å)
50	3500	1.882	1.968	1.957
135	4200	-2.577	8.525	1.796

Supplementary Table 3. Abundances of primordial noble gas nuclides in the core. ^{3}He budget in the bulk silicate Earth (BSE) is from nebular ingassing model that assumes a 10 Myr accretion time and 1 yr mean surface age[18]. Abundances of ^{22}Ne, ^{36}Ar and ^{130}Xe in BSE were calculated based their relative concentration to ^{3}He in ocean island basalts[19]. The ^{78}Kr abundance in BSE was calculated according to its relative abundance to ^{130}Xe in the whole Earth[20]. Abundances of the other nuclides were obtained by calibrating to their isotope solar ratios[21].

Nuclides	Differentiation conditions	Abundance (Pg, 1×10^{15} g)	
		BSE	Core
^{3}He	50 GPa, 3500 K	600	0.750
	135 GPa, 4200 K		0.248
^{20}Ne	50 GPa, 3500 K	3.32×10^{3}	0.003
	135 GPa, 4200 K		0.001
^{22}Ne	50 GPa, 3500 K	2.67×10^{3}	0.002
	135 GPa, 4200 K		0.001
^{36}Ar	50 GPa, 3500 K	2.06×10^{4}	0.018
	135 GPa, 4200 K		5.464
^{38}Ar	50 GPa, 3500 K	4.09×10^{3}	0.004
	135 GPa, 4200 K		1.086
^{78}Kr	50 GPa, 3500 K	1.56	2.355×10^{-5}
	135 GPa, 4200 K		4.987×10^{-3}
^{80}Kr	50 GPa, 3500 K	10.5	1.577×10^{-4}
	135 GPa, 4200 K		3.340×10^{-2}
^{82}Kr	50 GPa, 3500 K	56.0	8.434×10^{-4}
	135 GPa, 4200 K		0.179
^{83}Kr	50 GPa, 3500 K	0.310	4.672×10^{-6}
	135 GPa, 4200 K		9.893×10^{-4}
^{84}Kr	50 GPa, 3500 K	283	4.260×10^{-3}
	135 GPa, 4200 K		0.902
^{86}Kr	50 GPa, 3500 K	0.489	7.355×10^{-6}
	135 GPa, 4200 K		1.557×10^{-3}
^{124}Xe	50 GPa, 3500 K	23.6	0.122×10^{-3}
	135 GPa, 4200 K		0.417
^{126}Xe	50 GPa, 3500 K	11.7	0.110×10^{-3}
	135 GPa, 4200 K		0.378
^{128}Xe	50 GPa, 3500 K	0.568	2.263×10^{-3}
	135 GPa, 4200 K		7.761
^{130}Xe	50 GPa, 3500 K	0.627	4.574×10^{-3}
	135 GPa, 4200 K		15.687

Supplementary References

1 Boom, R., Kamperman, A. A., Dankert, O. & Van Veen, A. Argon solubility in liquid steel. *Metallurgical and Materials Transactions B* **31**, 913-919, (2000).
2 Gao, F., Deng, H., Heinisch, H. L. & Kurtz, R. J. A new Fe–He interatomic potential based on ab initio calculations in α-Fe. *Journal of Nuclear Materials* **418**, 115-120, (2011).
3 Li, Y., Korzhavyi, P. A., Sandström, R. & Lilja, C. Impurity effects on the grain boundary cohesion in copper. *Physical Review Materials* **1**, 070602(R), (2017).
4 Li, Y., Vočadlo, L., Sun, T. & Brodholt, J. P. The Earth's core as a reservoir of water. *Nature Geoscience* **13**, 453-458, (2020).
5 Guillot, B. & Sator, N. Noble gases in high-pressure silicate liquids: A computer simulation study. *Geochimica et Cosmochimica Acta* **80**, 51-69, (2012).
6 Schmidt, B. C. & Keppler, H. Experimental evidence for high noble gas solubilities in silicate melts under mantle pressures. *Earth and Planetary Science Letters* **195**, 277-290, (2002).
7 Kresse, G. & Furthmüller, J. Efficiency of ab-initio total energy calculations for metals and semiconductors using a plane-wave basis set. *Comput. Mater. Sci.* **6**, 15-50, (1996).
8 Kresse, G. & Hafner, J. *Ab initio* molecular dynamics for open-shell transition metals. *Phys. Rev. B* **48**, 13115, (1993).
9 Kresse, G. & Joubert, D. From ultrasoft pseudopotentials to the projector augmented-wave method. *Phys. Rev. B* **59**, 1758, (1999).
10 Blöchl, P. E. Projector augmented-wave method. *Phys. Rev. B* **50**, 17953, (1994).
11 Perdew, J. P., Burke, K. & Ernzerhof, M. Generalized gradient approximation made simple. *Physical Review Letters* **77**, 3865-3868, (1996).
12 Sun, T., Brodholt, J. P., Li, Y. & Vočadlo, L. Melting properties from ab initio free energy calculations: Iron at the Earth's inner-core boundary. *Physical Review B* **98**, 224301-224301, (2018).
13 Vočadlo, L. & Alfè, D. Ab initio melting curve of the fcc phase of aluminum. *Physical Review B* **65**, 1-12, (2002).
14 González-Cataldo, F., Wilson, H. F. & Militzer, B. Ab initio free energy calculations of the solubility of silica in metallic hydrogen and application to giant planet cores. *The Astrophysical Journal* **787**, 79, (2014).
15 Xiong, Z., Tsuchiya, T. & Van Orman, J. A. Helium and argon partitioning between liquid iron and silicate melt at high pressure. *Geophysical Research Letters* **48**, e2020GL090769, (2020).
16 Wang, K., Lu, X., Liu, X., Zhou, M. & Yin, K. Partitioning of noble gases (He, Ne, Ar, Kr, Xe) during Earth's core segregation: A possible core reservoir for primordial noble gases. *Geochimica et Cosmochimica Acta* **321**, 329-342, (2022).
17 Bennett, N. R. in *Encyclopedia of Geology (Second Edition)* (eds David Alderton & Scott A. Elias) 20-43 (Academic Press, 2021).
18 Olson, P. & Sharp, Z. D. Hydrogen and helium ingassing during terrestrial planet accretion. *Earth and Planetary Science Letters* **498**, 418-426, (2018).
19 Moreira, M. A. & Kurz, M. D. in *The Noble Gases as Geochemical Tracers* (ed Pete Burnard) 371-391 (Springer Berlin Heidelberg, 2013).

20 Halliday, A. N. The origins of volatiles in the terrestrial planets. *Geochimica et Cosmochimica Acta* **105**, 146-171, (2013).
21 Anders, E. & Grevesse, N. Abundances of the elements: Meteoritic and solar. *Geochimica et Cosmochimica Acta* **53**, 197-214, (1989).
